# Fabrication of Co-Doped Covalent Organic Framework Nanosheets with Mild Interlayer Stress for Quantitative Detection of Alzheimer’s Disease Biomarkers

**DOI:** 10.3390/bios16050271

**Published:** 2026-05-08

**Authors:** Yubing Lv, Yanli Zhou, Zi Liu, Hui Dong, Hejie Zheng, Sihan Cheng, Xu Wang, Chaoran Lv, Maotian Xu

**Affiliations:** 1 Henan Key Laboratory of Biomarker Detection and Diagnosis for Neurodegenerative Diseases, College of Chemistry and Chemical Engineering, Shangqiu Normal University, Shangqiu 476000, China; 2 Department of Hematology, Affiliated People’s Hospital of Jiangsu University, Zhenjiang 212000, China

**Keywords:** AD biomarkers, Co-TPCOF, co-catalysis, NADH, glutamate

## Abstract

Alzheimer’s disease (AD) seriously affects human health worldwide. Nicotinamide adenine dinucleotide (NADH) and glutamate are important biomarkers of AD, which play an indispensable role in the pathogenesis of AD. Herein, two ligands were used to synthesize a layered covalent organic framework (TPCOF) via amide bond formation, which was loaded with cobalt to obtain Co-TPCOF. TPCOF has a twisted 2D layered structure, along with large interlayer spacing and porosity, enabling precise Co coordination and stable loading of metal nanoparticles/enzymes to support electrocatalysis. A Co-TPCOF was immobilized on a screen-printed electrode (SPE) to catalyze the oxidation of NADH. After that, the oxidation product NAD^+^ of NADH and the NAD^+^-dependent dehydrogenase immobilized on the electrode jointly catalyzed the glutamate in the solution. COFs’ unique structures endow Co-TPCOFs with excellent NADH catalytic activity. The Co-TPCOF/SPE showed good linearity for NADH (10 nM-5 mM, LOD 7.07 nM) and GDH/Co-TPCOF/SPE for glutamate (50 μM-5 mM, LOD 3.74 μM). The biosensor can sensitively detect trace NADH and glutamate in human serum, providing an adequate technical means and theoretical reference for the pathological research of AD.

## 1. Introduction

The world’s population is ageing at an unprecedented rate, leading to the onset of various chronic diseases [1,2,3]. Alzheimer’s disease (AD) is a degenerative disease of the central nervous system that occurs with ageing. It is the most common form of dementia, with a progressive and chronic course, and a subtle onset [4]. AD places a significant socio-economic burden on both their families and governments. To maintain the quality of life for older individuals, intervention with biological mechanisms and control of the development of chronic diseases is an effective and urgently needed tool when AD becomes more severe.

Advancements in molecular diagnostics allow for accurate biomarker detection and quantification to evaluate disease onset or progression. Acceptable pathogenetic features of AD are the deposition of β-amyloid proteins that aggregate into extracellular plaques and the hyperphosphorylated tau proteins that misfold into intracellular neurofibrillary tangles [5,6,7]. It is hypothesized that these proteins accumulate abnormally in the brain 10–20 years before the onset of the disease in patients [8]. However, these proteins can indicate the severity of the disease rather than the initial change from ageing to disease [9]. Here, glutamate and nicotinamide adenine dinucleotide (NADH) levels in the body’s central nervous system were concurrently proposed as functional biomarkers, as they play an essential role in the pro-cognitive changes (cognitive change period) that precede the onset of mild cognitive impairment and eventual AD [10,11,12,13,14,15]. Inconsistent levels of glutamate and NADH in AD individuals may reflect pathological progression [12,14]. However, the evaluation of glutamate and NADH levels to identify the transition from healthy to dysfunctional ageing has not been reported.

Determination of glutamate and NADH in vivo and in vitro to understand their roles in neurotransmission remains challenging [15,16,17]. Electrochemical methods are promising due to their simplicity, rapidity, sensitivity, and specificity. NADH oxidation at electrodes is attractive, especially in biosensing. However, traditional electrochemical sensing strategies have limitations such as poor stability, long response time, poor reproducibility, and high electrode potential requirements [18,19,20,21]. Biocompatible nanomaterials are becoming popular due to their low cost in modified working electrodes. They facilitate electron transfer between enzymes and the electrode surface, and the high conductivity and increased electrochemically active surface area of nanomaterials enhance the catalytic activity of the electrode. Nanomaterials can lower the overpotential required for the electrochemical reaction, leading to more efficient sensing [12,14]. The development of new biocompatible nanomaterials is a positive and effective step towards progress. Metal–organic frameworks, especially zeolitic imidazolate frameworks, are unsuitable for body environment monitoring due to their chemical instability and structural collapse in acidic and alkaline environments [18,19,20,21,22,23,24,25].

A covalent–organic framework (COF) consists of two organic monomers linked by covalent bonds, and among the various linkages, the imine-bonded COFs have remarkable stability [23,26,27,28,29,30,31]. Among the various morphologies of COFs, a two-dimensional (2D) COF is suitable for the construction of electrochemical analytical sensors because of their large specific surface areas. Co^2+^ ions exhibit strong coordination with nitrogen-containing ligands, which are known for their excellent electrocatalytic properties [32]. The incorporation of Co^2+^ into the TPCOF structure enhances the electrochemical performance of the electrode, particularly in redox reactions relevant to our sensing applications. It is crucial for improving the sensitivity and selectivity of the electrochemical biosensor. However, 2D COFs accumulate on the electrode surface, affecting the detection results [33,34]. To overcome these drawbacks, 2,6-pyridinecarboxaldehyde (PCBA) and 5,10,15,20-tetrakis(4-aminophenyl)porphyrin (TAPP) were chosen as the ligand molecules for the synthesis of TAPP-PCBA COFs (TPCOFs) [34]. The nonlinear bridging ligands distort the planar connections between the porphyrin rings, weaken the planar stacking between the π-π porphyrin rings, and form contorted two-dimensional layers, which create significant gaps and more considerable interlayer distances, thus significantly reducing stacking interactions between the layers [35,36,37,38,39]. More importantly, the above COF could load abundant catalytic materials such as metal and metal oxide nanoparticles in the interlayer, which further enhanced its catalytic activity. However, the coordination of N to Co has a natural advantage over other coordinating atoms. The N-rich environment contained in our prepared COF could give a precise coordination environment for Co atoms. In addition, compared with previously reported porphyrin-based COFs, the PCBA ligand is an electron donor with fast electron transfer capability to the porphyrin ring, thus improving the overall electron transfer efficiency of the material. Porphyrin with an electron-deficient structure and a PCBA-cooperative COF system are ideal models for studying Co-COF catalysis.

**Scheme 1:**
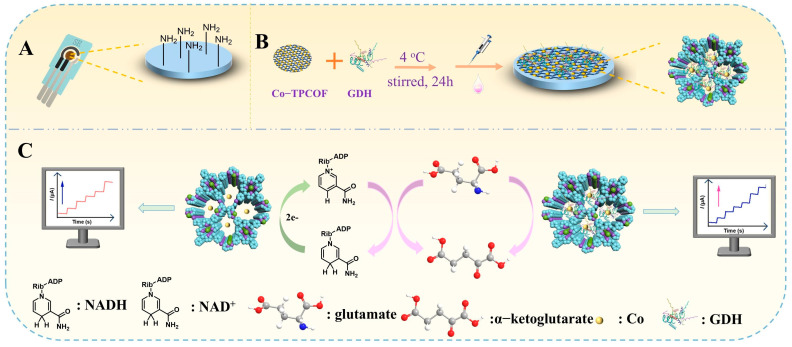
(**A**) The amino-modified SPE, (**B**) process scheme for preparing GDH/Co-TPCOF/SPE, (**C**) schematic representation of the catalysis of NADH and glutamate by Co-TPCOF and GDH/Co-TPCOF.

This study constructed an amperometric sensor using Co-loaded TAPP-PCBA COFs (Co-TPCOFs) as electrocatalytic nanomaterials to detect glutamate and NADH. COFs were directly metalized by heat-assisted refluxing to obtain a Co-TPCOF [40,41,42,43,44,45,46]. The modification of a Co-TPCOF on amino-functionalized screen-printed electrodes (SPEs) was based on covalent bonds, which avoided the excessive accumulation of nanomaterials on the surface of the electrodes. The enzyme adsorbed in the COF improves the stability of the enzyme and prevents its inactivation under non-ideal conditions. At the same time, the nanopore structure of the COF helps to maintain the natural conformation of the enzyme, thus maintaining its catalytic activity. Here, a “two-in-one” strategy is used, where Co and glutamate dehydrogenase (GDH) are co-anchored in the pores of an olefin-conjugated TPCOF, which builds an integrated platform based on a synergistic catalytic strategy (Scheme 1). This step-by-step nanomaterial–enzyme system exhibits excellent operational selectivity and stability, and integrates the detection of two AD-related biomarkers in a single platform, making it highly suitable for rapid and reliable serum analysis.

## 2. Experiment Section

### 2.1. Materials

Co(OAc)_2_⋅4H_2_O (AR), double ligands including PCBA, and TAPP were obtained from Kylpharma (Shanghai, China). NADH, NAD^+^, and GDH were supplied by Sangon Biotech Co., Ltd. (Shanghai, China). 1,2-Dichlorobenzene, AcOH, and Tetrahydrofuran were analytically pure. A traditional three-electrode system was used to carry out electrochemical measurements using a platinum wire as the counter electrode and Ag/AgCl saturated with 3 M KCl as the reference electrode. SPE was purchased from Redmatrix China Limited (Beijing, China). Unless otherwise stated, all other reagents and solvents were analytically pure and obtained commercially. The water involved in the experiment was purified by the Milli-Q pure water meter.

Characterization instruments were described in detail in Supporting Information.

### 2.2. Synthesis of the Co-TPCOF Composites

Co-TPCOF was prepared using the method described in previous reports, with some minor adjustments [34,45,47,48]. TAPP (81.3 mg, 0.12 mmol) and PCBA (32.4 mg, 0.24 mmol) were added to the mixed solution of ethanol (0.3 mL), 1,2-Dichlorobenzene (2.7 mL) and AcOH (6 M, 0.9 mL) in a Pyrex tube (15 mL) to form a mixture. The mixture was sonicated for 25 min, followed by immediately freezing the tube at 77 K (liquid N_2_ bath), repeated freezing and pumping three times. At this time, the tube was degassed to achieve an internal pressure of about 100 mTorr. The tube was heated at 120 °C for five days after the temperature returned to room temperature. Samples were filtered and transferred to a Soxhlet extractor to be washed with tetrahydrofuran (24 h) and acetone (24 h) after the tube was cooled to room temperature. Finally, the product was vacuumed at 120 °C for 24 h to obtain an activated sample (89.2 mg, ~88.5% yield).

A total of 100 mg of the above products was dissolved in 50 mL ethanol solution in a round bottom flask, and Co(OAc)_2_⋅4H_2_O (200 mg) was added with sonication for 2 min and the suspension was refluxed in a water bath overnight after cooling to room temperature, filtering and washing with water and ethanol thrice. The product was dried under vacuum overnight at 80 °C.

### 2.3. Fabrication of the Sensor Platforms

For the loading studies, 2 mg of the Co-TPCOF was first sonicated in 2 mL of the phosphate buffer (pH 8.0) for 10 min to get a homogeneous dispersion. GDH was immobilized on the synthesized support Co-TPCOF through physical adsorption. The buffer solution was degassed three times, treated with 2 mg of GDH and stirred at 4  °C for 24 h to absorb the enzyme into the pores of the COF. Immediately, the electrode was dried at 60 °C under vacuum for 6 h before the use of GDH/Co-TPCOF. Then, 7 µL of the solution sample was drip-coated on the SPE electrode directly. The electrode was dried by nitrogen and 7 µL of Nafion was added dropwise to form a film. The GDH/Co-TPCOF/SPE biosensor was fabricated after washing with PBS and stored at 4 °C to avoid denaturing.

### 2.4. Detection of Substances by Sensors

The concentration of NADH was determined by measuring the i-t curves at +0.52 V using the CHI760C electrochemical workstation. Different concentration solutions of NADH were added to the buffer solution. A 10 mM PBS buffer (pH 7.0) was used as the buffer solution and recorded for 200 s with a time resolution of 0.1 s while applying +0.52 V detection potential until the steady state was reached. The standard curve was obtained based on the relationship between steady-state current and the NADH concentration.

The amperometric i-t detections of glutamate by the GDH/Co-TPCOF/SPE biosensors were carried out at a potential of +0.65 V in PBS (50 mM, pH 8.0) containing 10 mM NAD^+^ added onto the modified working electrode surface. An aliquot of glutamate stock solution was added to the buffer solution at regular intervals. Amperometric i-t responses of the GDH/Co-TPCOF/SPE to injected substrate solutions were recorded. All the electrochemical experiments were performed at room temperature.

### 2.5. Analysis of Serum Samples

The i-t response of NADH in human serum was recorded by dropping different volumetric actual samples into the buffer solution at a potential of +0.52 V. At the same time, the i-t response of the glutamate in human serum was also recorded by adding different volumetric actual samples in PBS (50 mM, pH 8.0) containing 10 mM NAD^+^ at a potential of +0.65 V. All detection experiments for accurate sample analysis using the GDH/Co-TPCOF/SPE biosensors were performed at room temperature. If the concentrations of the target substances to be measured in the samples exceeded the detection limit, the samples could be diluted appropriately.

## 3. Results and Discussion

### 3.1. Design Strategy of the Electrochemical Sensor

Scheme 1A,B illustrates the proposed approach for the preparation of the GDH/Co-TPCOF/SPE. The Co-TPCOF was synthesized through a one-pot method and was utilized as a modifier to construct an NAD^+^-dependent dehydrogenase sensing platform. The GDH/Co-TPCOF-modified SPE (GDH/Co-TPCOF/SPE) was constructed to facilitate electron transfer between the catalytically active surface and the analyte for NADH monitoring. The process is shown in Scheme 1C. The NADH product was involved in the catalysis of glutamate, which accelerated the oxidation of glutamate. What is more, the NADH product participates in the reaction as a reactant in the next process, avoiding the accumulation of intermediate products on the electrode surface and the passivation of the electrode. Furthermore, the GDH of the constructed GDH/Co-TPCOF further catalyzes the glutamate substrate indirectly. The catalytic process of glutamate consumes NAD^+^, which is then regenerated through the oxidation of NADH. This creates a cyclic process where the two reactions are coupled and mutually reinforce each other, leading to a more efficient overall conversion. The proposed sensing strategy in the AD pathology hypothesis based on co-catalysis is expected to allow for the determination of two reliable markers.

### 3.2. Structural Characterization of the Co-TPCOF

**Figure 1:**
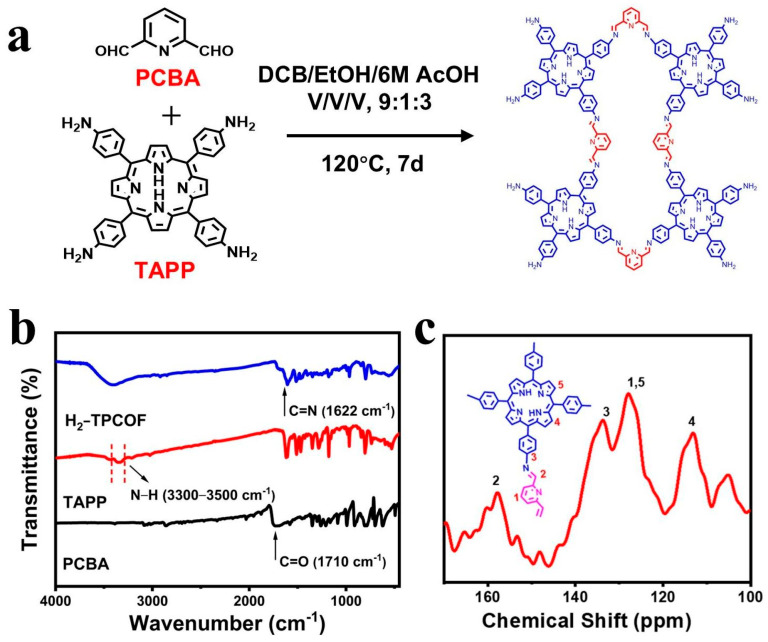
(**a**) Schematic representation of the synthesis procedure and structure; (**b**) FT–IR spectrum of TPCOF; (**c**) solid-state ^13^C NMR spectrum of TPCOF.

The chemical structure, crystallinity, and porosity of the prepared Co-TPCOF were characterized using various analytical techniques in Figure 1. Firstly, the N-containing imine-linked TPCOF was prepared under traditional solvothermal conditions by the condensation of TAPP and PCBA catalyzed by acetic acid (Figure 1a). The imine-based TPCOF material was obtained as a fuscous powder with a higher isolated yield and was not soluble in water or common organic solvents such as dichloromethane, acetone, and tetrahydrofuran. Additionally, Fourier-transform infrared (FT-IR) affirmed the chemical structures as synthesized. Concretely, the almost disappearances of the C=O characteristic vibration bond (1710 cm^−1^) and the N-H stretching vibration bond (3300–3500 cm^−1^) were probed in Figure 1b. At the same time, a new absorption peak near 1622 cm^−1^, for the characteristic vibration of the C=N bond, was observed, which showed the existence of imine linkages of the materials. Moreover, ^13^C solid-state nuclear magnetic resonance (^13^C NMR) spectroscopy indicates the chemical bond formation of the C=N bond, as evidenced by the appearance of signals at around 158 ppm in Figure 1c.

**Figure 2:**
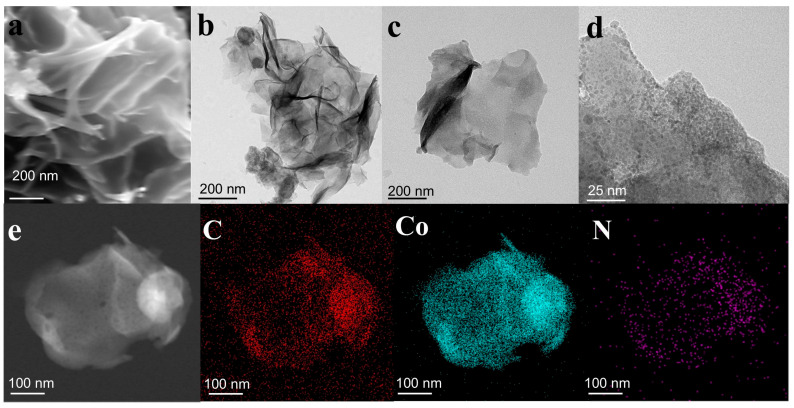
(**a**) SEM image of TPCOF; (**b**–**d**) TEM image of Co-TPCOF; (**e**) EDS mapping of C, Co, and N elements in selected area of Co-TPCOF.

Scanning electron microscopy (SEM) imaging analysis revealed that the morphology of TPCOFs were coral-like flakes with hundreds of nanometers (Figure 2a), which is different from that of the classic COF-366 (nanoparticle, about 100 nm). Transmission electron microscopy (TEM) images showed that the synthesized Co-TPCOF remained a wrinkled surface (Figure 2b) in the form of dispersed flakes (Figure 2c), and the surface of the flakes was uniformly loaded with Co nanoparticles (Figure 2d). The elemental distribution of energy dispersive X-ray spectra (EDSs) shows that the C, N, and Co elements are evenly distributed throughout the structure of the Co-TPCOF, which was consistent with expected results (Figure 2e). It can be seen from the Table S1 results of the EDS that the atomic percentage of element C is the highest (54.32%), and the atomic percentage of element N is 14.73%, which are mainly derived from the organic ligands PCBA and TAPP that construct the COF skeleton. The atomic percentage of element Co is 4.70%, which confirms that Co species have been successfully loaded into the TPCOF structure, consistent with the Co 2p characteristic peak results observed in the XPS analysis. In addition, the detection of element O (26.24%) may be related to the oxidation on the material surface or oxygen-containing functional groups in the ligands. The XPS spectrum analysis in this paper also indicates that slight surface oxidation may occur during the synthesis process. These data further verify the successful preparation of the Co-TPCOF composite material.

**Figure 3:**
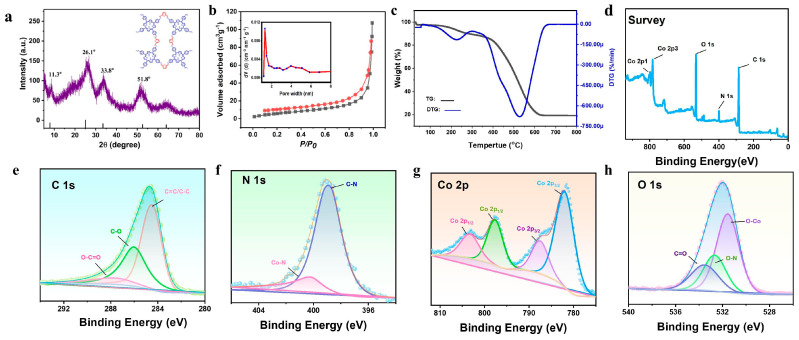
(**a**) Experimental PXRD patterns; (**b**) N_2_ sorption isotherm curve of TPCOF measured at 77 K and inset is the pore-size distribution profile; (**c**) TGA curves of TPCOF under air atmosphere; (**d**) XPS profile of Co-TPCOF; (**e**) high-resolution N 1s XPS profiles of Co-TPCOF; (**f**) high-resolution C 1s XPS profiles of Co-TPCOF; (**g**) high-resolution Co 2p XPS profiles of Co-TPCOF; (**h**) high-resolution O 1s XPS profiles of Co-TPCOF.

Additionally, the crystal structures of the TPCOF were resolved by using powder X-ray diffraction (PXRD) measurements (Figure 3a). In the PXRD pattern, the peak signals at 11.3° are assigned to the (600) facets. A diffraction peak at 2θ = 22.78° was ascribed to the conjugation stacking of 2D layers [44]. The interlayer spacing is calculated to be 0.389 nm according to Bragg’s law (see the supporting literature for details). This result is consistent with the typical range of π-π stacking distances, which verifies the interlayer stacking mode. Based on the fined structure, the TPCOF had a peanut-like structure and was expected to be a mesoporous material. The accessible porosity of the resulting material was studied on the porosimetry analyzer through N_2_ adsorption experiments at 77 K on the micromeritics surface area (Figure 3b), and the samples were degassed and activated at 120 °C for 12 h before testing. The adsorption isotherm of the sample analysis by nonlocal density functional theory using a cylindrical pore model showed that the pore size distribution was 1.3 nm, and the surface Brunauer–Emmett–Teller (BET) of the TPCOF was calculated as 537.4 m^2^ g^−1^, which was very consistent with the expected structure [35].

To prove the thermal stability of TPCOFs, a continuous thermogravimetric analyzer (TGA) was used to study its thermal behavior (Figure 3c). The heating range was room temperature to 800 °C and the heating rate was 10 °C/min under the protective atmosphere of nitrogen. As shown, the temperature points with the fastest decomposition rate for the sample are 400–600 °C. From the first derivative of the thermogravimetric curve, the TPCOF was almost pyrolyzed in nitrogen at 600 °C. X-ray photoelectron spectroscopy (XPS) measurements demonstrated the elements and chemical bonds of polymer material coordination of Co-TPCOFs. In the range of 0 to 800 eV, the typical peaks of Co 2p, C 1s, N 1s, and O 1s were observed (Figure 3d). In Figure 3e, the N 1s spectra have appeared at 401.5, 399.8 eV, which is affiliated with N-Co, C=N. The C 1s spectra of the Co-TPCOF had three characteristic peaks located at 288.4 eV, 286.3 eV, and 284.7 eV, which corresponded to the groups of O-C=O, C-O, and C-C/C=C, respectively (Figure 3f). The observed binding energy of Co 2p_3/2_ was 782.1 eV and the binding energy of 2p_1/2_ is 798.2 eV, which is assigned to Co^2+^ and indicates that the Co ion was successfully modified into the structure (Figure 3g). The results showed that the Co-TPCOF could contain small amounts of Co species impurities (such as CoO and metal Co) [45]. Additionally, some oxidation may occur during Co-TPCOF synthesis on the surface of O 1s (Figure 3h). The elemental stoichiometric ratio of the Co-TPCOF is shown in Table S2. The XPS test results are highly consistent with the EDS analysis results, confirming the successful introduction of cobalt active sites and that the overall elemental stoichiometric ratio of the Co-TPCOF meets the design requirements. The slight discrepancy between the XPS and EDS results may stem from the fact that XPS is a surface-sensitive analytical technique, while EDS focuses on the bulk characterization of materials. This difference further verifies the uniform distribution of elements inside the material. The TPCOF was successfully synthesized and was post-metalized by Co nanoparticles.

### 3.3. Process Analysis for Biosensor Manufacture

**Figure 4:**
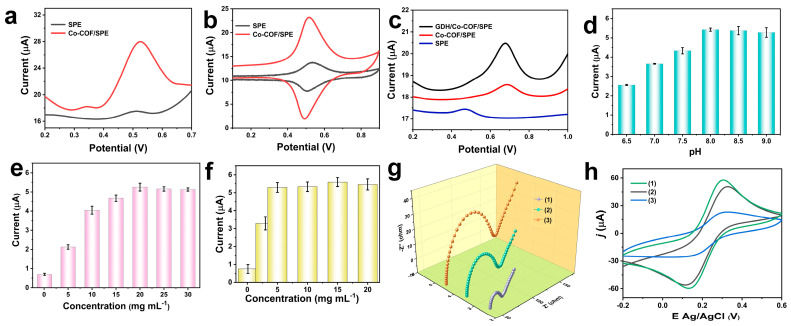
DPV (**a**) and CV (**b**) curves of SPE and Co-TPCOF/SPE modified electrodes in 10 mM PBS (pH 7.0, the concentration of NADH was 5 mM); (**c**) DPV curves of GDH/Co-TPCOF/SPE, Co-TPCOF/SPE and SPE modified electrodes in 50 mM PBS (pH 8.0, the concentration of glutamate was 3 mM); (**d**) effects of pH in buffer solution; (**e**) GDH concentration in the label preparation; (**f**) Co-TPCOF concentration in the label preparation; EIS (**g**) and CV (**h**) analysis of different modified electrodes: (1) bare SPE, (2) GDH@Co-TPCOF/SPE, (3) after immobilization of Nafion.

The Co-TPCOF was used as a modifying material to construct an NAD^+^-dependent dehydrogenase sensing platform to facilitate electron transfer between catalytically active surfaces and analytes for NADH monitoring. In PBS with a pH of 7.0 and a concentration of 10 mM, the DPV responses of the bare SPE and Co-TPCOF/SPE were observed in the presence of NADH. The results shown in Figure 4a indicated that the bare SPE did not exhibit any redox peak in the potential window from 0.2 V to 0.7 V, while the Co-TPCOF/SPE showed a clear oxidation peak potential at +0.52 V (*E*_pa_). This indicates that the Co-TPCOF/SPE exhibits a significant catalytic effect on NADH, while the bare SPE does not. Directly immobilization of the enzyme onto the electrode surface without Co-TPCOF conditions would be a technical challenge, with small amounts of the enzyme immobilized on the electrode surface, mostly in the free enzyme form. It is well-established that the Sp. Of the activity of glutamate dehydrogenase is 9 ± 0.33, and immobilized enzymes perform much better electrochemically than free enzymes. The immobilization method using the Co-TPCOF as a molecular binder to the SPE surface successfully produced a stable enzyme layer on the electrode surface.

In addition, the electrochemical preparation process was characterized by analyzing the oxidation peak current intensity versus scan rate (ν) at *E*^0^ = 0.52 V (Figure S1). The linear correlation between the Co-TPCOF/SPE current intensity and the square root of the scan rate suggests that the electrochemical process is not significantly hindered by electron transfer kinetics. This agrees with the theory because the Co-TPCOF was immobilized on the surface of the SPE working electrode by amination [45,46], which avoided its stacking and loss on the electrode surface. The CV curves of the SPE and Co-TPCOF/SPE in 5.0 mM NADH (pH 7.0) were shown in Figure 4b. While both the bare SPE and Co-TPCOF/SPE could be electro-oxidized, the Co-TPCOF/SPE showed a significantly higher oxidation peak. This enzyme is commonly used for catalyzing the deamination of amino acids by glutamate, especially the oxidative deamination of glutamate to alpha-ketoglutarate in the presence of NAD^+^. The NAD^+^-dependent dehydrogenase of the NADH oxidation product efficiently oxidized a wide range of substrates, with the catalytic effect on glutamate as demonstrated in Figure 4c. Glutamate dehydrogenase was adsorbed in the Co-TPCOF nanopore structure, which acts together with NAD^+^ to catalyze the generation of glutamate and its derivatives and catalyze the conversion between a pair of cofactors (NAD^+^ and NADH).

*NADH* → *NADH^+^* + *e^−^* (1)

*NADH^+^* → *NAD^−^* + *H^+^* (2)

*NAD^·^* → *NAD^+^* + *e^−^* (3)

*glutamate* + *NAD^+^* + *H_2_O* ↔ *α-ketoglutarate* + *NADH* + *NH_4_^+^* + *H^+^* (4)

In addition, the enzymatic reaction was studied by controlling the ratio of NAD^+^ to NADH to be 1:1, which reduced the reaction products’ effect on enzyme inhibition and achieved more efficient cofactor regeneration. In sensing and monitoring systems, the performance of the sensing element is crucial for the biosensor’s overall performance, and it is an essential part of sensor preparation. Therefore, enzyme concentration, Co-TPCOF concentration, and buffer solution pH were systematically investigated to obtain more accurate, sensitive, and stable biosensors for co-catalyzed systems. Figure 4d shows the effect of buffer solution pH on the performance of the electrochemical biosensor. pH tests were conducted within the range of 6.5–9.0. The experimental findings for glutamate detection revealed that the optimum signal current response was achieved at pH 8.0. This is because extremely acidic or alkaline environments can cause biomolecules, such as proteins, to become inactive. The NAD^+^ concentration (4 mM) and substrate concentration (3 mM) were kept constant, and the effect of enzyme loading of the sensor’s glutamate dehydrogenase on catalytic efficiency was recorded at room temperature (Figure 4e). At a glutamate dehydrogenase concentration of 20 mg mL^−1^, the electrochemical response reached a steady state, the enzyme loading reached saturation, and a further increase in loading led to a marginal decrease in the effect. Similarly, the optimal concentration of the Co-TPCOF was 5 mg mL^−1^ (Figure 4f). During the fabrication process, CV and electrochemical impedance spectroscopy (EIS) were used to evaluate the electrode surface’s electrochemical redox behavior. CV characterization was carried out in a 0.1 M KCl solution containing 2.5 mM [Fe(CN)_6_]^3−/4−^ (Figure 4h). EIS characterization was carried out in a solution containing 5 mM [Fe(CN)_6_]^3−/4−^ (Figure 4g). It can be seen that the surface of the electrodes was modified sequentially with the addition of the GDH/Co-TPCOF and Nafion; the addition of non-electrically active substances resulted in a weakening of the electron transfer capacity at the electrode surface.

### 3.4. Analytical Performances

**Figure 5:**
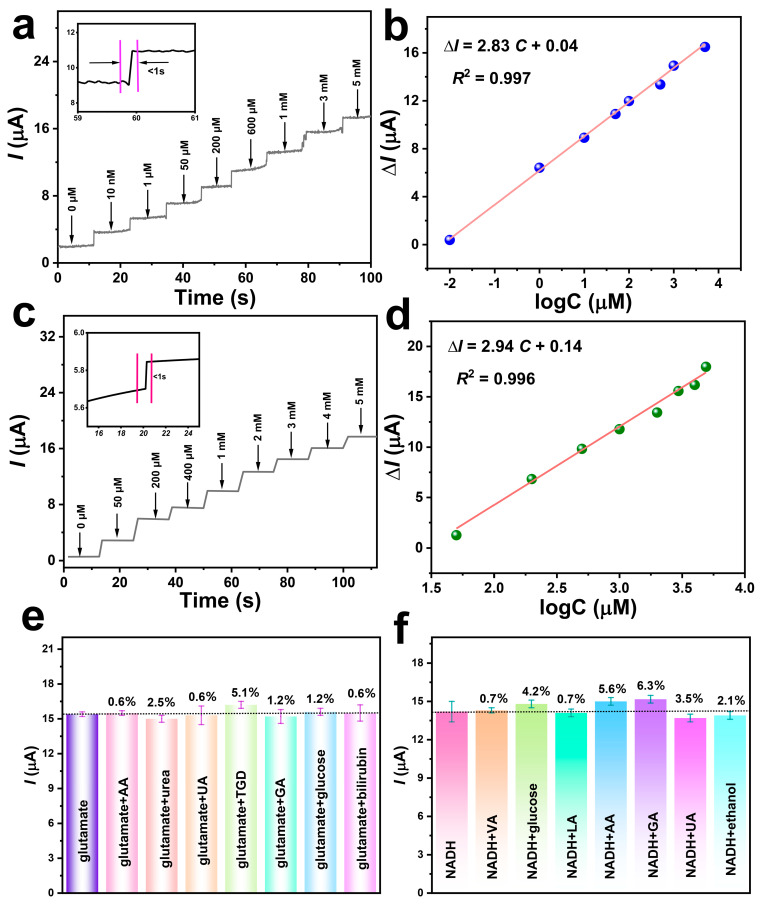
(**a**) Amperometric i-t responses of the GDH/Co-TPCOF/SPE upon successive additions of NADH to PBS (10 mM, pH 7.0). The inset profile shows the response time; (**b**) calibration curves between the current change and concentration of NADH; (**c**) amperometric i-t responses of the GDH/Co-TPCOF/SPE upon successive additions of glutamate to PBS (50 mM, pH 8.0); the inset profile shows the response time; (**d**) calibration curves between the current change and concentration of glutamate; (**e**) selectivity of the glutamate in PBS (50 mM, pH 8.0) containing 1 mM of various interference species (glutamate, 3 mM); (**f**) selectivity of the NADH in PBS (10 mM, pH 7.0) containing 1 mM of various interference species (NADH, 3 mM).

The i-t curves of different concentrations of NADH and glutamate were recorded with the prepared sensor under optimal conditions. The steady-state current response at a potential +0.52 V with continuous addition of NADH was shown in Figure 5a. From 10 nM to 5 mM, Δ*I* (current difference between signal and blank) was proportional to the logarithmic value of concentration for NADH. The linear relationship conformed to the linear regression equation Δ*I* = 2.83 log*C* + 0.04 (*R*^2^ = 0.997) (Δ*i*_p_ = *i*_p_ − *i*_p0_) (Figure 5b). The limit of detection was calculated to be 7.07 nM (S/N = 3). Different concentrations of NADH showed equally good DPV responses in the system (Figure S2). The high catalytic capacity of COFs may be the reason for the superior performance of the effect compared to other reported biosensors based on electrooxidation sensors of NADH (Table S3). In addition, the sensing system has a quick signal response to the analyte, with a steady state reached in under 0.1s. Different pH buffers were used to monitor glutamate oxidation rates as NAD^+^-dependent dehydrogenase functions best in an acidic environment. In the range of 50 μM to 5 mM, with the glutamate concentration increases, a linear relationship between the current and logarithmic value of concentration was established as Δ*I* = 2.94 log*C* + 0.14 (*R*^2^ = 0.997) with a low detection limit of 3.74 μM (Figure 5c,d). The COF’s strong immobilization of the enzyme, biocompatibility, and co-catalytic strategy with NAD^+^ gave a competitive advantage to the assay for glutamate over the previous examples (Table S4).

The i-t test was repeated six times with the same modified electrode sensor for NADH and glutamate at a concentration of 3 mM, and the RSD values of the current response were 0.8% and 1.3%, respectively (Figure S3). Using six different electrodes to repeatedly test the same NADH and glutamate concentration, their current responses had relative standard deviations of 8.9% and 7.1%, respectively. The long-term stability of the sensor was satisfactory, as the current decreased by 20% after 35 days (Figure S4). The stability of interference is an important factor in determining whether a sensor has been constructed successfully. The presence of redox-active interfering substances (e.g., ascorbic acid, uric acid, glucose, and other amino acids) in the environment is a major concern because of the environment where AD occurs includes cerebrospinal fluid and serum. To avoid errors in the results, the i-t current responses of NADH and glutamate were recorded separately in the presence of a 1 mM interference group as shown in Figure 5e,f. The proposed sensor displays superior selectivity as none of the results leading to a change in the current response exceeded 7% even with the addition of a high concentration of the possible interference group. Therefore, the GDH/Co-TPCOF/SPE sensor exhibits high selectivity against common interferences and shows some long-term stability at room temperature.

### 3.5. Real Sample Analysis

To explore the clinical analytical ability of the constructed sensor in real samples and reveal diagnostic value for AD, we collected clinical serum samples from the Affiliated Hospital of Jiangsu University including five AD patients and five non-AD individuals. Small amounts of vitamin A and coenzyme Q were added at the first time to stabilize NADH in the serum from oxidation. Additionally, 1 mM EDTA was added into the solution to minimize interference from metal ions (e.g., Cu^2+^) [49]. The electroanalytical test results are presented in Table 1, after diluting the prepared samples. The concentration of NADH in the human body ranges from 0.05 to 500 μM, as indicated by reports [50,51]. The NADH concentration range analyzed using this sensor aligns well with previous reports. Table 1 shows the results of the analysis of serum glutamate content using the GDH/CO-TPCOF/SPE. We showed that the serum glutamate amount was significantly higher in AD patients compared to non-AD patients, but NADH did not significantly increase when compared to non-AD patients [52]. Intriguingly, glutamate and NADH serum levels appeared to be positively correlated with AD. The GDH/CO-TPCOF/SPE has preliminary applicability in detecting glutamate and NADH in real samples. Overall, our data suggest that small molecule metabolites in blood could lead to accurate metabolomic profiling of AD, and unveil novel diagnostic and prognostic biomarkers. These two indicators are functional auxiliary biomarkers rather than definitive diagnostic markers, and their abnormal levels only suggest the risk of cognitive impairment and energy metabolism disorders related to the pathological process of AD.

## 4. Conclusions

In summary, an electrochemical biosensor was fabricated based on a synergistic catalytic strategy for the determination of trace NADH and glutamate in human serum. In detail, a biocatalytic sensing platform based on GDH/Co-TPCOF was successfully constructed, and this strategy has several remarkable features compared with reported methods. Firstly, the chemical inertness of the surface of the two-dimensional material was overcome by using asymmetric bridging ligands to distort the ligand connections, thereby reducing the detrimental effects of stacking between planes. Secondly, the sensing performance was greatly improved by the Co metallization of a COF, which was one of the most reliable carrier nanomaterials for Co nanoparticles. It can flexibly modulate sensing performance, reduce the overpotential of NADH oxidation, have a low detection limit, and respond quickly. Additionally, the co-catalysis of NAD^+^-dependent glutamate dehydrogenase and an oxidation product of NADH achieved sensitive detection of glutamate and showed high specificity and sensitivity. This novel sensing method enables one-step simultaneous detection of two key AD biomarkers with superior reproducibility, stability, and accuracy compared with most conventional biosensors. This work is expected to provide valuable references for promoting AD-related biomedical research, facilitating early clinical diagnosis, and supporting other relevant studies. This approach provides valuable references for promoting biomedical research related to AD, early clinical diagnosis, and other relevant studies.

## Figures and Tables

**Table 1:** Determination of the NADH and glutamate from human serum samples by GDH/CO-TPCOF/SPE.

	Sample	NADH Content (μM)	ELISA (μM)	*t*	Glutamate Content (μM)	ELISA (μM)	*t*
Normal	1	1.08 ± 0.02	1.21	1.42	10.3 ± 0.73	10.11	2.77
	2	1.04 ± 0.02	1.19	0.55	5.4 ± 0.13	5.12	1.94
	3	1.88 ± 0.03	1.61	1.76	5.7 ± 0.16	5.49	0.21
	4	2.17 ± 0.06	1.68	0.98	11.4 ± 0.15	10.88	1.82
	5	2.62 ± 0.06	2.60	1.45	16.7 ± 0.33	16.65	1.47
Patient	1	2.33 ± 0.04	2.46	1.58	20.5 ± 1.21	21.08	1.64
	2	1.12 ± 0.03	1.37	0.77	33.9 ± 0.80	31.75	1.36
	3	2.87 ± 0.08	3.22	1.54	21.8 ± 0.72	22.03	0.33
	4	1.81 ± 0.04	1.65	2.21	34.2 ± 0.77	33.14	0.32
	5	3.12 ± 0.02	3.26	0.47	31.2 ± 0.86	30.06	0.33

## Data Availability

The original contributions presented in this study are included in the article. Further inquiries can be directed to the corresponding authors. Clinical trial number: [2025]KY016-01.

## Supplementary Materials

The following supporting information can be downloaded at: https://www.mdpi.com/article/10.3390/bios16050271/s1, Figure S1: Calibration curve of oxidation peak value vs. square root of the scan rate of the process; Figure S2: DPV responses of the proposed electrochemical strategy with different concentrations of NADH, the range of concentration is 10 nM to 5 mM; Figure S3: (a) The reproducibility and (b) The repeatability evaluation of the electrochemical assay for 3 mM NADH in 10 mM PBS (pH 7.0) and 3 mM glutamate in 50 mM PBS (pH 8.0); Figure S4: (a) Shows the sensor’s Long-term storage stability (the concentration of NADH concentrations was 5 mM), and (b) Shows the sensor’s Long-term storage stability (the concentration of glutamate concentrations was 5 mM); Table S1: Total atomic distribution spectrum; Table S2: XPS analysis of Co-TPCOF; Table S3: Performance of various types of NADH sensors; Table S4: Performance of various types of glutamate sensors. References [53,54,55,56,57,58,59,60,61,62,63,64,65] are cited in the Supplementary Materials.
