# Supplementary material for: Fabrication of Co-Doped Covalent Organic Framework Nanosheets with Mild Interlayer Stress for Quantitative Detection of Alzheimer’s Disease Biomarkers
Source: Biosensors (Basel). 2026 May 8;16(5):271. doi: 10.3390/bios16050271 (PMC13204613; doi:10.3390/bios16050271)
Supplement: Supplementary file 1 [file biosensors-16-00271-s001.zip › biosensors-4253343-Supplementary.pdf]

Fabrication of Co-Doped Covalent Organic Framework Nanosheets with Mild Interlayer Stress for Quantitative Detection of Alzheimer's Disease Biomarkers

Yubing Lv[a], Yanli Zhou[a,*], Zi Liu[a], Hui Dong[a], Hejie Zheng[a], Sihan Cheng[a], Xu Wang[a], Chaoran Lv[b,*], Maotian Xu[a]

[a] *Henan Key Laboratory of Biomarker Detection and Diagnosis for Neurodegenerative Diseases, College of Chemistry and Chemical Engineering, Shangqiu Normal University, Shangqiu, 476000, Henan, China*

[b] *Department of Blood Diseases, Institute of Jiangsu University, Affiliated People's Hospital of Jiangsu University, Zhenjiang, 212000, Jiangsu, China*

***Corresponding authors.**

E-mail addresses: zhouyanli@mails.ucas.ac.cn (Y. Zhou); lcran2011@163.com (C. Lv)

Characterization and instruments

Fourier transform infrared spectroscopy (FT-IR) was collected on a Bruker Tensor in the range of 4000-400 cm^{-1} using the technique of pressed KBr pellets. ^{13}C CP/MAS solid-state nuclear magnetic resonance (^{13}C-NMR) spectra were obtained from Bruker AVANCE III 400 spectrometer. Scanning electron microscopy (SEM) was performed using a SU8020 field emission at an accelerating voltage of 3.0 kV with an EMAX energy-dispersive X-ray analyzer (Horiba, USA). Transmission electron microscopy (TEM) images and high-resolution TEM (HR-TEM) images were collected on an FEI Tecnai G2 F30 (USA) electron microscope at 200 kV equipped with an Oxford Energy dispersive X-ray spectroscopy. The PXRD spectra were recorded on a Bruker D8 Advance X-ray diffractometer (Germany) with Cu Kα radiation (λ = 1.5418 Å) at 45 kV, 200 mA. Nitrogen sorption tests were performed at 77 K using a Micromeritics ASAP 2020 volumetric adsorption analyzer. TGA tests were carried out on a Diamond TG/DTA/DSC Thermal Analyzer System (Perkin-Elmer, USA). X-ray photoelectron spectroscopy (XPS) was recorded using an Escalab 250Xi instrument (Thermo Scientific) equipped with an Al Kα microfocused X-ray source, and the C1s peak at 284.8 eV as the internal standard.

All electrochemical measurements were carried out using a CHI660E electrochemical workstation (Shanghai Chenhua Instrument Co., Ltd.). In the three-electrode system, all potentials were referenced to an Ag/AgCl electrode, with the SPE as the working electrode and the platinum wire as the auxiliary electrode. Impedance

measurements were performed in a 0.1 M KCl solution containing 5 mM $[Fe(CN)_6]^{3-/4-}$, with an AC potential of 10 mV in the frequency range from 0.1 Hz to 0.1 MHz. Cyclic voltammetry at a scan rate of 50 mV s^{-1}. The volt-ampere response signal of the sensor was recorded with a pulse amplitude of 0.004 V, pulse width of 0.1 μs, and sample width of 0.0167 μs, over the potential range of 0 V to 1 V.

Table S1 Total atomic distribution spectrum

Total distribution spectrum							
Element	Line Type	k Factor	k Factor Type	Absorption Correction	Wt%	Wt% Sigma	Atomic Percentage
C	K-series	2.369	Theoretical	1.00	20.93	0.31	54.32
N	K-series	2.998	Theoretical	1.00	10.00	0.17	14.73
O	K-series	1.718	Theoretical	1.00	32.53	0.30	26.24
Co	K-series	1.000	Theoretical	1.00	36.54	0.28	4.70
Total：					100.00		100.00

Table S2 XPS analysis of Co-TPCOF

Name	Start BE	Peak BE	End BE	Height CPS	FWHM eV	Area (P) CPS.eV	Area (N) TPP-2M	Atomic %
C1s	296.28	284.75	280.58	34263.81	2.79	114771.02	1609.31	60.18
N1s	409.18	398.96	394.08	6334.7	2.41	19500.31	176.1	6.59
O1s	541.88	532.02	527.18	35954.54	3.03	117821.59	683.57	25.56
Co2p	812.38	781.97	772.88	18201.39	5.16	178642.95	205.21	7.67

Calculate the interlayer spacing by applying Bragg's Law of COF (d):

The mathematical formula of Bragg's law is: $n\lambda = 2d \sin\theta$, where n represents the diffraction order,λ is the wavelength of incident X-rays, d denotes the interplanar

spacing of crystal planes, and θ stands for the Bragg angle. Cu Kα radiation source is commonly used in XRD testing, with a wavelength of approximately 0.154 nm. Substitute the values for calculation: d≈0.39 nm.

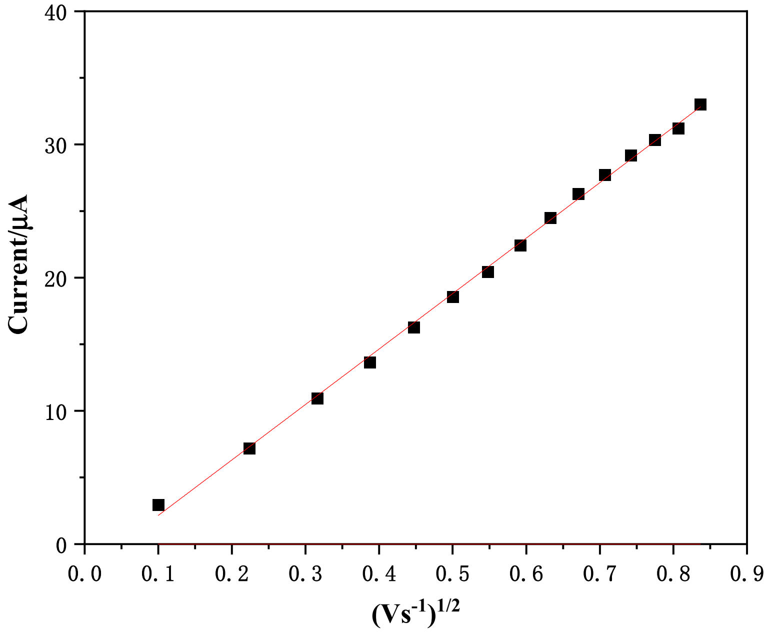

Fig. S1 Calibration curve of oxidation peak value vs. square root of the scan rate of the process

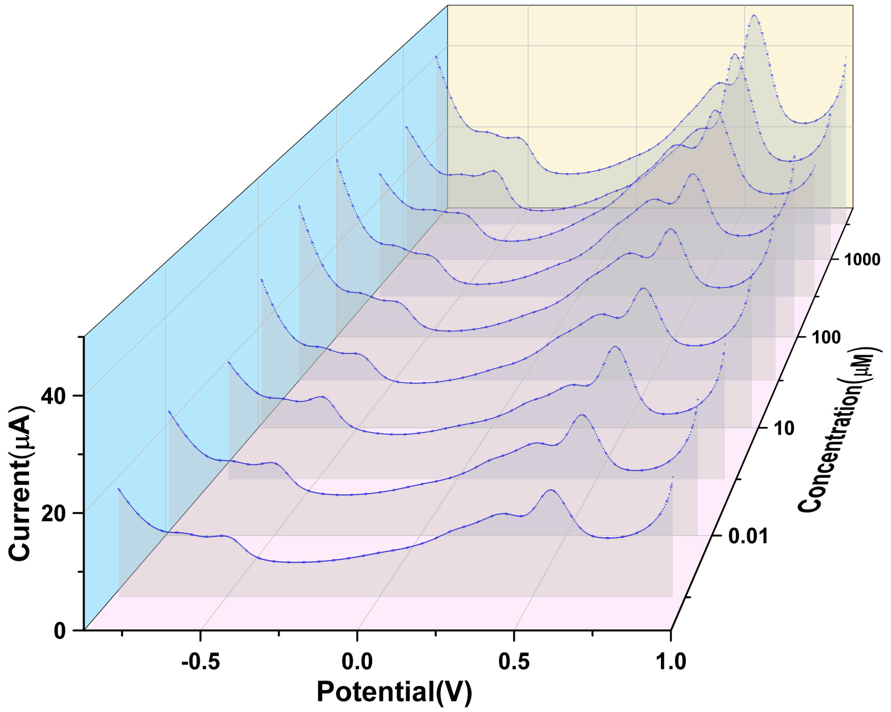

Fig. S2 DPV responses of the proposed electrochemical strategy with different

concentrations of NADH, the range of concentration is 10 nM to 5 mM.

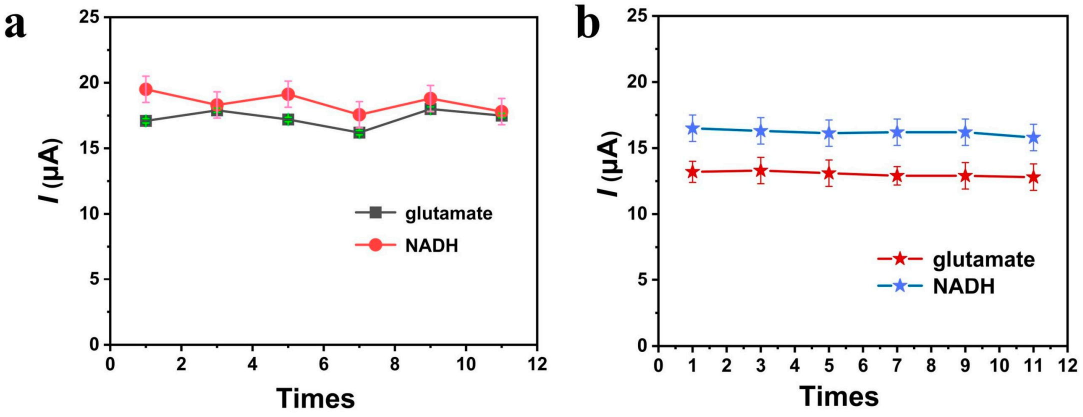

Fig. S3 a) The reproducibility and b) The repeatability evaluation of the electrochemical assay for 3 mM NADH in 10 mM PBS (pH 7.0) and 3 mM glutamate in 50 mM PBS (pH 8.0).

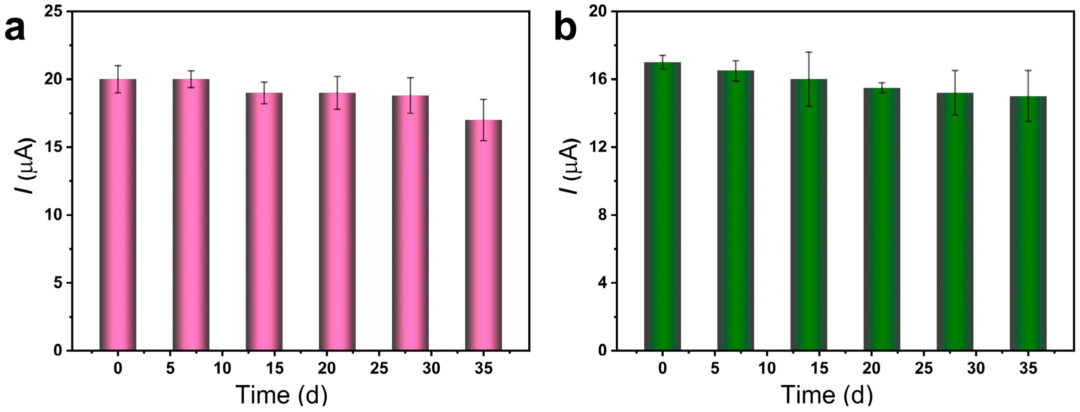

Fig. S4 a) Shows the sensor's Long-term storage stability (the concentration of NADH concentrations was 5 mM), and b) Shows the sensor's Long-term storage stability (the concentration of glutamate concentrations was 5 mM).

Table S3 Performance of various types of NADH sensors.

Sensing platform	Detection element	Technique/Working Potential (V)	Detection range (μM)	Limit of detection (μM)	Reference
Au/MB/graphene	NADH	Amp./+0.74	1-264	0.3	53
GCE/MWCNT/poly-FA	NADH	Amp./+0.20	59.1-1560	17.73	54
IL/graphene/chitosan/GCE	NADH	Amp./+0.45	250-2000	5	55
GC/MWCNT/1-AP	NADH	Amp./+0.10	1-210	0.08	56
MnO_2/CuS-$BiVO_4$/ITO	NADH	-	$0.005\text{-}10\times10^{-3}$	3.38×10^{-3}	57
PAH/PMo11V_4/GCE	NADH	Amp./+0.6	3.2-850	0.19	58
Au@PDA/TiC/GCE	NADH	Amp./+0.6	5-450	3.17	59
Co-COF/SPE	NADH	Amp./+0.52	0.01-5000	7.07×10^{-3}	This study

Table S4 Performance of various types of glutamate sensors.

Sensing platform	Enzyme	Operating voltage (V)	Detection range (μM)	Limit of detection (μM)	Reference
GCE/Naf/GDH-bacteria/PEI-MWNT	GDH	0.70	10-1000/2000-10000	2	60
SPCE/MB/chit/MWCNT	GDH	0.10	7.5-105	3	61
Pt-MEA/BSA/GluALD/mPD	GluOx	0.70	10-570	0.16±0.02	62
Pt/PoPD/AscOx/BSA	GluOx	0.60	5-150	0.044	63
GLDH/NPG/GCE	GDH	0.40	50-700	6.82	64
GLDH/Chit-AA-CDs/SPCE	GDH	0.00	0.5-8000	3.3	65
GDH/Co-COF/SPE	GDH	0.65	50-5000	3.74	This study

1. Erarkc, E.; Bayndr, O.; Alanyalolu, M. Amperometric quantification of NADH based on graphene/methylene blue nanocomposite thin films on Au(111). *Polym. Compos.* **2017**, *38*, E118–E127.
2. Da Silva, L.V.; Lopes, C.B.; Da Silva, W.C. Electropolymerisation of ferulic acid on multi-walled carbon nanotubes modified glassy carbon electrode as a versatile platform for NADH, dopamine and epinephrine separate detection. *Microchem. J.* **2017**, *133*, 460–467.
3. Shan, C.; Yang, H.; Han, D. Graphene/AuNPs/chitosan nanocomposites film for glucose biosensing. *Biosens. Bioelectron.* **2010**, *25*, 1070–1074.
4. Amanda, L.; Tomasz, R.; Robert, F.; Teofil, J.; Grzegorz, M. Electrochemical generation of 1-amino-pyrene-4,5,9,10 tetrol on the MWCNT surface for low potential electrocatalytic NADH oxidation. *Electrochim. Acta* **2023**, *463*, 142822.
5. Gao, L.; Zhou, Y.; Cao, L.; Cao, Y.; Zhang, H. Photoelectrochemical sensor for histone deacetylase Sirt1 detection based on Z-scheme heterojunction of CuS-$BiVO_4$ photoactive material and the cyclic etching of MnO_2 by NADH. *Talanta* **2024**, *268*, 125307.
6. Chu, M.; Bai, Z.; Zhu, D.; Chen, W.; Yang, G.; Xin, J.; Ma, H. Aβ-nicotinamide adenine dinucleotide electrochemical sensor based on polyoxometalate built by the combination of electrodeposition and self-assembly. *Electroanal. Chem.* **2022**, *907*, 116083.
7. Vusa, C.; Gokhale, N.; Panda, S. Electro-Structured Cu distorted nanopyramids for superior sweat glucose sensin. *Food Chem.* **2023**, *426*, 136609.
8. Liang, B.; Zhang, S.; Lang, Q.; Song, J.; Han, L.; Liu, A. Amperometric L-glutamate biosensor based on bacterial cell-surface displayed glutamate dehydrogenase. *Anal. Chim. Acta* **2015**, *884*, 83–89.
9. Hughes, G.; Pemberton, R.M.; Fielden, P.R.; Hart, J. A Reagentless, Screen-Printed amperometric biosensor for the determination of glutamate in food and clinical applications. *Sens. Actuators B. Chem.* **2015**, *216*, 614–621.
10. Scoggin, J.L.; Tan, C.; Nguyen, N.H.; Kansakar, U.; Madadi, M.; Siddiqui, S.; Arumugam, P. U.; DeCoster, M.A.; Murray, T.A. An enzyme-based electrochemical biosensor probe with sensitivity to detect astrocytic versus glioma uptake of glutamate in real-time in vitro. *Biosens. Bioelectron.* **2019**, *126*, 751–757.
11. Ganesan, M.; Trikantzopoulos, E.; Maniar, Y.; Lee, S.T.; Venton, B.J. Development of a novel micro biosensor for in vivo monitoring of glutamate release in the brain. *Biosens. Bioelectron.* **2019**, *130*, 103–109.
12. Cai, T.; Shang, K.; Wang, X.; Qi, X.; Liu, R.; Wang, X. Integration of glutamate dehydrogenase and nanoporous gold for electrochemical detection of glutamate. *Biosensors* **2023**, *13*, 1023.
13. Martinez-Perinan, E.; Dominguez-Saldana, A.; Villa-Manso, A.M. Azure A embedded in carbon dots as NADH electrocatalyst: Development of a glutamate electrochemical biosensor. *Sens. Actuators B. Chem.* **2023**, *374*, 132761.
